# GENDER-Q Youth Patient-Reported Outcome Measure: Protocol for an International Cross-Sectional Field Test Study

**DOI:** 10.2196/100420

**Published:** 2026-10-05

**Authors:** Anne F Klassen, Charlene Rae, Shane D Morrison, Natasha Johnson, Karine Khatchadourian, Jack Graesser, Manraj N Kaur

**Affiliations:** 1 Department of Pediatrics, Faculty of Health Sciences, McMaster University, 3N27, 1280 Main Street West, Hamilton, ON, L8N3Z5, Canada, 1 905-521-2100 ext 73775; 2 Department of Surgery, Division of Plastic Surgery, University of Washington Medical Center, Seattle, WA, United States; 3 Department of Pediatrics, University of Ottawa, Ottawa, ON, Canada; 4 Department of Clinical Research, University of Southern Denmark, Odense, South Denmark, Denmark; 5 Department of Surgery, Harvard Medical School, Patient-Reported Outcomes, Values & Experience Center (PROVE), Mass General Brigham, Boston, MA, United States

**Keywords:** gender-affirming care, youth, hormones, mixed methods, patient-reported outcomes, protocol

## Abstract

**Background:**

The need for gender-affirming care (GAC) for young people has increased rapidly worldwide. GAC includes social, psychological, and medical interventions aimed at alleviating gender-related distress. Given that many outcomes of GAC relate to how young people function and feel, a rigorously developed patient-reported outcome measure (PROM) is needed. To address this need, GENDER-Q Youth was developed with extensive input from youth with lived experience. Concept elicitation interviews were performed with 47 youth from Canada and the United States. A conceptual framework and draft PROM were developed and refined with feedback from 33 experts and 17 youth, and were pilot tested with 406 older youth. The field test version of GENDER-Q Youth includes 16 independently functioning scales (248 items) that measure health-related quality of life, gender practices, voice, and experience of care.

**Objective:**

This paper outlines the protocol for an international cross-sectional study to field test GENDER-Q Youth in a sample of transgender and gender diverse (TGD) adolescents and young adults aged 12 to 25 years receiving GAC.

**Methods:**

This study follows international guidelines for PROM development and validation (eg, US Food and Drug Administration and Consensus-Based Standards for the Selection of Health Measurement Instruments [COSMIN]). GENDER-Q Youth was translated into Danish, Dutch, and German following the Professional Society for Health Economics and Outcomes Research (ISPOR) guidelines for the translation and cultural adaptation of PROMs. For the field test, TGD youth are being recruited using multiple strategies (eg, face-to-face, emails, and so on). A REDCap survey is used to collect sociodemographic and clinical data. Branching logic is used to ensure relevant GENDER-Q Youth scales are completed as some scales are gender-specific. For psychometric analysis, Rasch measurement theory (RMT) analysis will be used to examine fit of the observed data to the Rasch model. A series of tests and criteria will examine item fit and scale reliability and validity. Test-retest reliability will be examined with intraclass correlation coefficients. For construct validity, 147 predefined hypotheses of expected group differences and correlations between GENDER-Q Youth scales will be examined. Acceptance of at least 75% of hypotheses is considered sufficient evidence of construct validity per COSMIN criteria.

**Results:**

The research described in this protocol is funded by a Canadian Institutes of Health Research Sex and Gender Science Chair (April 2020) and supported by a Canada Research Chair in Patient-Reported Outcomes (April 2025). With the Danish, Dutch, and German translations now completed, the international field test is underway in Canada, Europe, Australia, and the United States. Data collection and analysis are expected to be completed by the end of 2026.

**Conclusions:**

This protocol describes the international GENDER-Q Youth field test study. Once developed, GENDER-Q Youth can be used to inform GAC, research, and quality improvement efforts.

## Introduction

### Background

The global need for gender-affirming care (GAC) for young people has increased substantially, especially among those assigned female at birth [1]. GAC encompasses social, psychological, and medical interventions that aim to alleviate gender-related distress. Individual treatment goals vary but can include treatments that modify how the voice sounds and change how the face and body look and function. Studies have shown the benefits of GAC in terms of improved appearance congruence and mental health outcomes with low levels of regret [2-5]. Concurrently, systematic reviews of gender-affirming treatments for youth (eg, puberty blockers, hormones, and mental health care) suggest that long-term outcomes and overall quality of evidence remain limited [6-12].

Since many outcomes associated with GAC affect how people function and feel, rigorously developed patient-reported outcome measures (PROMs) specific to gender-diverse populations are needed. This need is underscored by 6 systematic reviews of PROMs, which converge to conclude that PROMs used in GAC are typically generic (for use in any health condition), designed for cisgender populations, and designed without adherence to international PROM development guidelines [13-18]. One review to date examined PROMs used with transgender and gender diverse (TGD) youth [18] identifying 38 measures that had been used in research with TGD youth. Most PROMs are generic measures of psychological function or quality of life. Such tools were not developed to measure outcomes of GAC and therefore lack content validity for TGD youth. Three gender-specific PROMs that measure gender-specific concepts were found, but published evidence about how these PROMs were developed and validated is lacking. The use of such PROMs in research with TGD youth can result in inaccurate or misleading conclusions by failing to ask the right questions [19]. Also missing are validated scales that measure how TGD youth experience their care [20]. Based on current literature, there is currently no rigorously developed comprehensive PROM that can be used to measure outcomes and experience of care for TGD youth.

To address the lack of validated GAC-specific PROMs, our team developed the GENDER-Q for adults. This comprehensive PROM includes 54 unidimensional scales and 2 checklists covering domains of health-related quality of life, sexual, urination, gender practices, voice, hair, face and neck, body, breasts, genital feminization, chest, genital masculinization, and experience of care [21,22]. GENDER-Q was designed to evaluate any form of GAC for adults, including all forms of gender-affirming surgery. To address the need for a PROM for TGD youth, our focus was to design a comprehensive set of scales to evaluate the multidisciplinary care provided in pediatric gender clinics. As such, GENDER-Q Youth does not include scales to measure surgical outcomes but instead focuses on measurement of outcomes related to social, psychological, voice, and medical treatments (eg, puberty blockers, hormones) provided to TGD youth by pediatricians and other healthcare providers.

Our study adheres to international guidelines for PROM development and validation, including the US Food and Drug Administration, the Consensus-Based Standards for the Selection of Health Measurement Instruments (COSMIN), and ISPOR (The Professional Society for Health Economics and Outcomes Research) Principles of Good Practice for the Translation and Cultural Adaptation of PROMs [23-28]. Figure 1 shows our mixed method approach, which is grounded in the experiences of TGD youth. Our team has completed step 1 of a mixed method study to identify the concepts that GENDER-Q Youth should measure. Full details for the sample, methods, and results for step 1 are published [29]. Briefly, we conducted concept elicitation interviews with 47 youth aged 12 to 19 years from Canada and the United States who were seeking or receiving GAC. Interviews were digitally recorded, transcribed, and coded. Analysis led to the development of a conceptual framework (Figure 2) and an item pool. The items were used to form a set of scales that were refined with input from 33 experts and 17 TGD youth. Content validity was further explored in an online sample of 120 youth aged 18 to 25 years.

**Figure 1.**
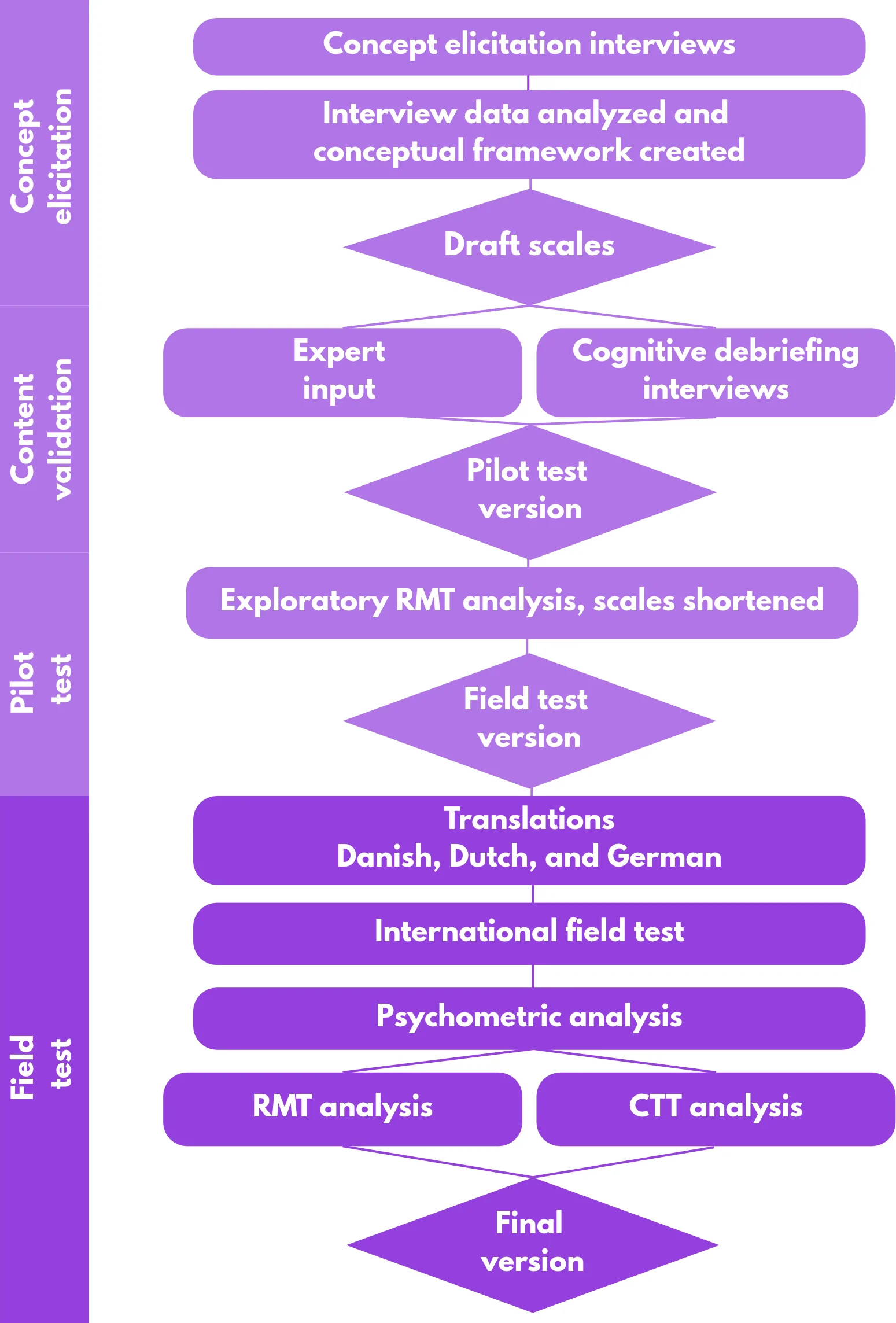
Methods diagram. CTT: classical test theory; RMT: Rasch measurement theory.

**Figure 2.**
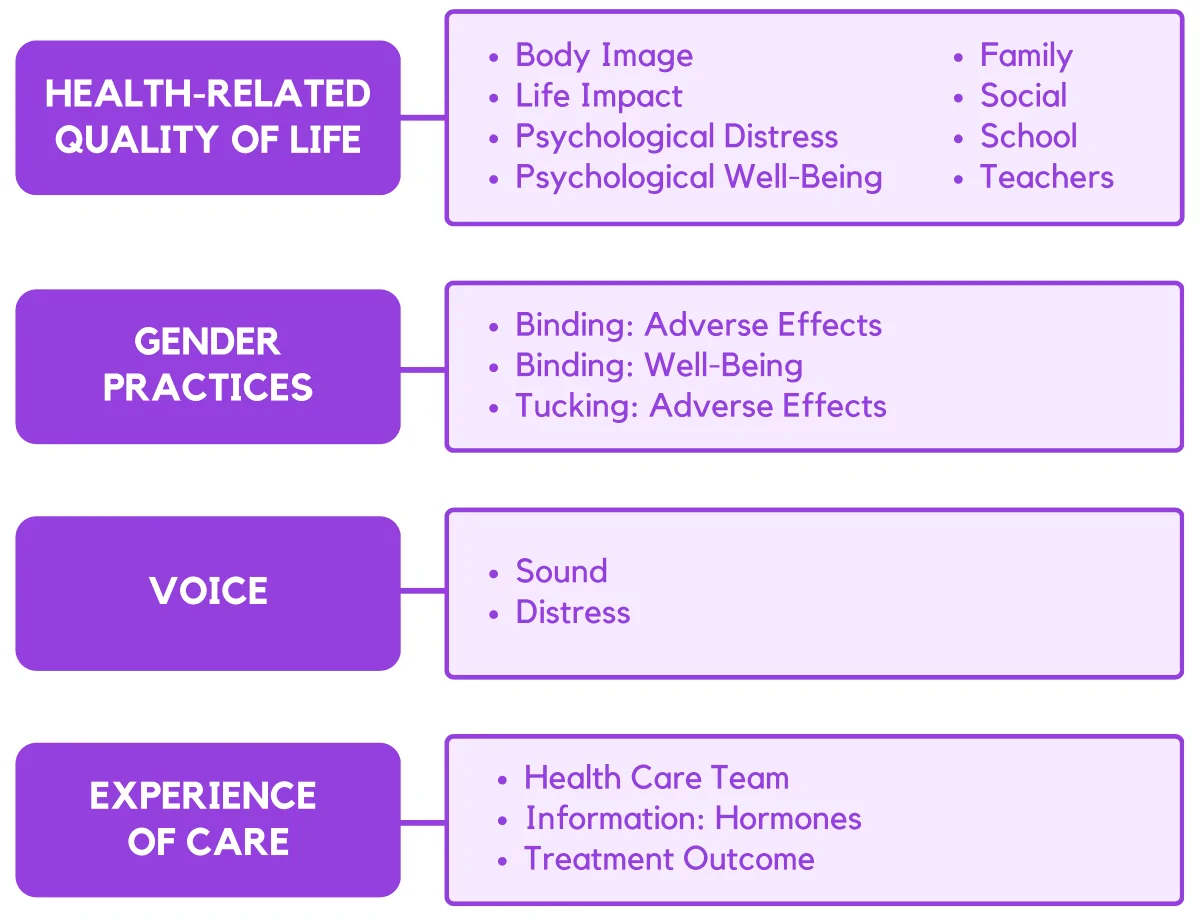
GENDER-Q Youth conceptual framework.

GENDER-Q Youth was pilot tested in an international sample of 406 youth aged 18 to 25 years recruited from the Prolific crowd-sourcing platform. The sample was also used to collect test-retest (TRT) data for GENDER-Q Youth scales. For psychometric analysis, Rasch measurement theory (RMT) analysis [30-33] was used to identify items with extreme misfit to the Rasch model (11 items were dropped from 7 scales). At the end of step 1, the field test version of GENDER-Q Youth included 16 scales (248 items). Concepts used to develop the items for each scale and example quotes to illustrate participants’ experiences are published elsewhere [29]. The Flesch-Kincaid Grade Level for GENDER-Q Youth field test scales ranged from 0.5 to 5.0.

### Objective

The objective of this study is to field test GENDER-Q Youth in a large international sample of TGD youth aged 12 to 25 years seeking or receiving GAC and to evaluate its psychometric performance in order to provide a comprehensive PROM for use in research, clinical care, and quality improvement.

This objective will be achieved by examining a range of measurement properties outlined by the COSMIN initiative [27,28]. To shorten each scale, we will conduct RMT analysis, a modern psychometric method that uses a probabilistic measurement model to identify the optimal set of items to retain in each scale. In this approach, selected items should measure the full range of the underlying construct with high reliability and validity. A series of statistical tests (eg, ordered thresholds, item fit, local dependency, and differential item functioning) will guide decisions about which items to retain. The resulting scales will be unidimensional, invariant, and easy to interpret. Additional classical test theory (CTT) tests will be performed to examine scale stability (ie, test-retest reliability) and construct validity by testing predefined hypotheses for group differences and correlations between GENDER-Q Youth scales.

## Methods

### Ethical Considerations

Research ethical approval has been obtained for the McMaster University coordinating site (Hamilton Integrated Research Ethics Board #17427). Ethical approvals and data sharing agreements will be obtained for all participating sites prior to data collection. In Canada, youth under the age of 18 years must complete an assent form and have a parent or primary caregiver provide consent for their participation in the study unless a clinical team member has deemed the individual capable of consenting for themselves. In all other countries, participants younger than 18 years of age must have a parent or primary caregiver provide consent for their participation unless a waiver of parental consent is obtained from their local ethics board. All participants aged 18 years or older may provide consent for themselves. Participant consent in the research study will be obtained in the REDCap survey (Vanderbilt University). The survey is created and hosted at McMaster University or a participating site’s server as per each institution’s requirements. In the reporting of results, the STROBE (Strengthening the Reporting of Observational Studies in Epidemiology) reporting guidelines for cross-sectional studies will be followed [34].

At the start and end of the survey, participants are provided with a list of local resources they can access for support if needed (eg, Trans Lifeline Canada and Kids Help Phone). The survey is designed such that participants are required to answer a limited number of questions needed for branching logic (eg, sex assigned at birth) to ensure they receive the appropriate items and scales (eg, scales measuring binding vs tucking). In addition, demographic and clinical questions include “Prefer to not answer” or “I want to skip this question” as response options. The rest of the survey is set up such that participants can skip any items or scales they are not comfortable answering. At the end of the survey, to measure the impact of the GENDER-Q Youth survey on participants, we ask how answering the survey made them feel (ie, “Very unhappy,” “A little unhappy,” “Same as usual,” “A little happy,” and “Very happy”). An open text box is provided for comments. The impact of completing GENDER-Q Youth in relation to demographic and clinical characteristics will be examined in the analysis.

### Translations

In preparation for the international field test, GENDER-Q Youth has been translated into Danish, Dutch, and German following the Professional Society for Health Economics and Outcomes Research (ISPOR) guidelines for PROM translation and cultural adaptation [26]. This approach involved two independent forward translations, reconciliation, back-translation into English, and cognitive debriefing interviews with at least 5 youth aged 12 to 25 years who represented different gender identities. Youth were asked to review the translated instructions, response options, and items to determine if they were understandable and culturally relevant. The goal was to develop conceptual and culturally equivalent translations of GENDER-Q Youth. Each translation team also translated the site-specific consent letter and REDCap survey (eg, demographic and clinical questions).

### Sample

The field test sample includes youth recruited in multiple countries. Eligible TGD youth are aged 12 to 25 years seeking or receiving GAC who are able to complete the survey independently in one of the GENDER-Q Youth translations. The upper age range is used since pediatric hospitals and gender clinics in some countries provide GAC to the young adult population.

### Recruitment

Participant recruitment uses a number of strategies and approaches to accrue the sample. In hospitals and clinics (gender, endocrinology, voice therapy, and/or surgery), participants are recruited in-person, virtually (eg, Zoom), by email or phone call, or through electronic medical record messaging (eg, MyChart). Youth who completed a concept elicitation interview in the GENDER-Q Youth step 1 study [29], and eligible participants from the GENDER-Q adult field test study who agreed to be recontacted [21], are invited to complete the field test survey. In the community, TGD youth aged 18 years and older are recruited via email and social media posts through 2SLGBTQI+ (Two-Spirit, lesbian, gay, bisexual, transgender, queer or questioning, and intersex) community groups, advocacy groups, and professional/clinical services organizations.

### Data Collection

An online REDCap questionnaire is used to collect self-reported demographics (eg, age, gender identity, race, education, and country), health status (eg, health conditions including neurodivergent diagnoses), and health care utilization variables including number of visits to the hospital or clinic for GAC; treatments participants have had or want to have in the future such as puberty blockers, hormones, voice or speech therapy, and mental health care and surgery. For gender identity, participants are first provided with an open text box with the question: “What word or words would you use to describe your gender?” This question is followed with the fixed choice question: “If you had to pick one of the following, would you say that you are” with answer options: “Boy or Male,” “Girl or Female,” “Nonbinary,” and “Another gender.” Participants that choose “Another gender” receive an open text box that asks: “Can you please tell us what other gender you identify as?” Participants are also asked the following question: “Do you identify as transgender?” Answer options include the following: “No,” “Yes,” and “Not sure.” After the gender questions, sex at birth is asked and used with branching logic to ensure that participants receive the correct gender practices scales (ie, for tucking or binding). This question is worded as follows: “What was your assigned sex at birth?” The options include the following: “Male,” “Female,” and “Not sure.” Those who select not sure are asked: “Are you intersex?.” Answers include: “No,” “Yes,” and “Not Sure.” Additional questions were asked to measure construct validity for GENDER-Q Youth scales. An example is the question “How happy are you with the people you see in the clinic (eg, doctor, nurse, social worker)?” Answers could be “Not happy,” “A little happy,” “Quite happy,” and “Very happy.” This question will be used to examine group differences for the hypothesis that scores would be higher on the GENDER-Q Youth Health Care Team scale for those who reported being happier.

The REDCap survey is hosted on McMaster University’s secure server or at collaborating sites if required by their institution. Youth in clinics have the option of completing the REDCap survey using an iPad provided in the clinic or their own device. Youth who are emailed a link to the survey can use their own device. Participants who complete the survey receive a US $18 gift card equivalent to thank them for their time if allowed by their local ethics board.

### Data Analysis

Sociodemographic and clinical characteristics for the study sample, and the question asking about the impact of completing the GENDER-Q Youth will be summarized using descriptive statistics. Data from the pilot test will be combined with the field test data for the following psychometric analyses.

#### RMT Analysis

##### Overview

RMT analysis is an established modern psychometric method used in rating scale design and psychometric evaluation [30-33]. This approach uses a probabilistic framework to model the relationship between a person’s latent trait level and item difficulty. Rasch analysis orders items hierarchically from hardest to easiest to endorse (“Rasch ruler”). When observed data fit the expectations of the Rasch model, the resulting scale supports invariant and interpretable interval-level measurement. RMT analysis will be used to examine how items function together within each scale and to identify redundant or poorly functioning items. Our goal is to identify a subset of items for each scale that best represents the underlying construct, resulting in shorter, clinically meaningful scales with strong measurement properties. Analyses will be conducted using RUMM2030 software (RUMM Laboratory) with the unrestricted partial credit model for polytomous data. We will examine the following item and scale properties described below.

##### Thresholds for Item Response Options

Category thresholds between adjacent response options (eg, “Never” and “Rarely”) will be examined to determine whether response categories function as they are intended. Ordered thresholds indicate whether participants with higher levels of the construct are more likely to endorse higher response categories. Disordered thresholds indicate that respondents may have trouble distinguishing between adjacent response categories. Items with disordered thresholds can be collapsed (rescored) to improve ordering or removed from the scale.

##### Item Fit Statistics

Three fit indicators will be examined: fit residuals, item characteristic curves, and chi-square statistics. Fit residuals are expected to fall within the range of −2.5 to +2.5, chi-square values should be nonsignificant after Bonferroni adjustment, and intraclass correlation curves should depict agreement between observed and expected responses. We will interpret fit statistics together and in relation to their clinical importance.

##### Dependency

We will examine residual correlations between item pairs to assess local item dependence. Residual correlations greater than 0.30 above the average residual correlation will indicate potential dependency, which can artificially inflate reliability [35]. For item pairs showing dependency, subtests will be created to evaluate their impact on scale reliability.

##### Targeting

We will examine the distribution of item locations relative to person locations to assess targeting. Good targeting is indicated when item thresholds are spread across the range of the construct and align with the distribution of participants. Poor targeting may result in floor and/or ceiling effects.

##### Differential Item Functioning

We will examine differential item functioning (DIF) to determine if subgroups (ie, gender, age, country, and language) respond differently to items despite having the same level of underlying trait. For gender, DIF will be examined by comparing three groups as follows: boy/male, girl/female, and nonbinary. Where subgroup sizes are unequal, random samples will be selected to create equal-sized groups. Items with chi-square values that are significant will be considered for deletion during item reduction. We will also examine the impact of DIF on each scale by splitting items on the variable demonstrating DIF, and correlating the person locations from the original and split solutions.

##### Reliability

We will compute the Person Separation Index for each scale to assess the reliability of each scale and the ability to distinguish between individuals of different levels of the trait for each scale. We will also compute Cronbach α to examine the internal consistency of items within each scale [36]. Reliability coefficients of >0.70 are considered acceptable [28].

##### Unidimensionality

The Rasch model requires that scales measure a single unidimensional construct [33]. This assumption can be tested using the Smith test [37]. In this test, items are split into 2 subsets based on a principal component analysis of the residuals and person estimates are compared. If fewer than 5% of tests show significantly different estimates between the 2 subsets, the scale is considered unidimensional [32].

### CTT Analysis

After the RMT analysis is completed, the item-reduced scales will be scored by transforming person locations into scores that range from 0 to 100. For all scales, higher scores will indicate a better outcome. The following psychometric tests will be performed.

#### TRT Reliability

We will examine TRT reliability in stable participants using a 2-way mixed effect model intraclass correlation coefficient for consistency. A reliability coefficient of >0.70 is considered acceptable [28].

#### Smallest Detectable Change

TRT data will also be used to compute the smallest detectable change (SDC) beyond measurement error at the individual and group levels [28]. Change scores greater than the SDC suggest real change rather than change due to chance. For a scale to be useful, the SDC should be smaller than the minimally important difference (MID) [28]. Calculating an MID value is beyond the scope of the current study.

#### Construct Validity

COSMIN guidelines informed our plan for construct validity testing [27,28,38]. Construct validity examines whether an instrument measures the construct it was designed to assess by comparing differences between groups and associations between scales. To this end, we created a set of questions to test the constructs measured by GENDER-Q Youth scales. These hypotheses examine a wide range of group differences as well as correlations. We will test hypotheses regarding group differences and correlations between GENDER-Q Youth scales. This approach to construct validity requires specification of both the direction and magnitude of predetermined hypotheses [27]. COSMIN considers construct validity adequate if at least 75% hypotheses are confirmed, although guidance on the number of hypotheses to test is lacking [28]. Multimedia Appendix 1 presents the predefined hypotheses for the GENDER-Q Youth study, with directionality specified for each hypothesis. We formulated 147 hypotheses (3-17 per scale) to test. The goal for construct validation is to examine the overall acceptance of the tests rather than focus on individual test results.

### Group Hypotheses

We will test 69 group hypotheses that use demographic information, treatment information, and Likert questions included in the survey. For dichotomous variables, mean differences with 95% CIs will be computed and interpreted using Cohen *d* (0.20 small, 0.50 medium, and 0.80 large) [39]. For variables with more than 2 response options, effect sizes will be calculated using η² with 95% CIs and interpreted as follows: <0.01 negligible, 0.01 ≤η²<0.06 small, 0.06 ≤η²<0.14 medium, and ≥0.14 large [39]. All group differences are hypothesized to demonstrate at least a small effect size (d≥0.2 or η²≥0.01).

### Correlational Hypotheses

We will test 78 correlational hypotheses. Associations between scales will be examined using Pearson correlation coefficients (*r*). For correlations between GENDER-Q Youth scales, COSMIN-based criteria are applied: scales measuring similar constructs are expected to correlate *r*≥0.5; scales measuring related but dissimilar constructs are expected to correlate 0.3 to <0.5, and scales measuring unrelated constructs are expected to correlate *r*<0.3 [39]. Some scales in the GENDER-Q adult module (ie, Body Image, Gender Dysphoria, Psychological Well-being, Psychological Distress, Social, Voice Sound, Voice Distress, and Treatment Outcome) measure similar constructs as scales in the youth module. For construct validation, the magnitude of correlations between GENDER-Q Youth scales is expected to be similar to the correlations between the 8 GENDER-Q adult module scales . For GENDER-Q Youth scales without an adult counterpart, we hypothesized scales would correlate 0.30, indicating the scales measure related constructs.

## Results

This study is funded by a Canadian Institutes of Health Research (CIHR) Sex and Gender Science Chair Award received in April 2020. This award provided grant funds to develop and field test GENDER-Q Youth with TGD youth receiving GAC in Canada and the United States. In April 2025, AFK was awarded a Tier 1 Canada Research Chair in Patient-Reported Outcomes. This award provided funds that made it possible to translate GENDER-Q Youth into multiple languages and expand the field test to include TGD youth in Europe, and Australia. Including youth in multiple countries makes it possible to capture a much broader range of experiences in the dataset that will be used to item-reduce each scale and develop the scoring algorithms.

Step 1, which included concept elicitation interviews, conceptual framework development, scale item generation, content validation, and pilot testing, has been completed [29,40,41]. Elsewhere we report that the sample of youth in the pilot testing found GENDER-Q Youth to be easy to understand (94%), thorough (92%), safe to complete (91%), and that it asked important questions (80%) in a respectful way (91%) [29].

In preparation for step 2, GENDER-Q Youth has been translated into Dutch, Danish, and German. Field testing is taking place at 11 participating sites in Canada (n=2), the United States (n=4), Denmark (n=1), the Netherlands (n=1), England (n=1), Germany (n=1), and Australia (n=1). We anticipate that data collection and analysis will be completed by the end of 2026 and we expect to publish results in the first half of 2027. Once GENDER-Q Youth is finalized, our team will examine psychometric properties for the item-reduced final scales as well as score interpretability.

## Discussion

### Principal Findings

There is a rising international need for GAC for TGD young people and for more high-quality evidence for the effectiveness of gender-affirming treatment. GENDER-Q Youth will provide a tool that researchers and clinicians can use to collect evidence-based outcomes data from youth aged 12 to 25 years who identify as TGD. In step 1, data from 47 youth were collected and analyzed to form a conceptual framework, items, and preliminary scales [29]. Content validity was demonstrated with feedback from 33 experts, and cognitive debriefing interviews with 17 youth [29]. In step 2, our international field test study will provide important psychometric evidence about the measurement properties of GENDER-Q Youth. Our study will item-reduce GENDER-Q Youth scales and provide evidence of reliability and validity. Such information is needed before GENDER-Q Youth can be used in research, clinical care, and health care policy. Validation is an ongoing process with evidence accumulating over time and across multiple studies. Future research will be needed to examine other measurement properties, such as responsiveness, and to enhance interpretability of GENDER-Q Youth scale scores by computing MIDs.

TGD people experience substantial barriers to accessing care and continue to have unmet health care needs [42]. Given the rising demand for GAC for TGD young people, and the limited evidence for the effectiveness of treatment, a robust comprehensive PROM is needed to capture the patient perspective in outcome assessment. GENDER-Q Youth will provide a rigorously developed and validated PROM that can be used in research studies and clinical care to evaluate outcomes that matter most to TGD youth. Once complete, the GENDER-Q Youth can be used in the evaluation of GAC or in clinics to compare outcomes across studies, monitor change in outcomes over time, as well as to inform shared decision-making. Gender-specific PROMs for youth are important as they can help inform evidence-based care and improve delivery of GAC.

### Strengths and Limitations

This study has strengths and limitations. A key strength is the meaningful patient involvement that is taking place throughout all stages of the development and validation of GENDER-Q Youth. Such input is essential to ensuring that GENDER-Q Youth measures concepts relevant to patients in language that is appropriate and respectful. Second, the inclusion of a large heterogeneous international sample that varies by age, gender identity, country, language, and treatment history will enable the capture of a broad range of experiences. Third, the use of a modern psychometric approach (RMT analysis) and modular design means that each scale is unidimensional and independently functioning, which will allow end users to choose only the most relevant scales for their purpose or needs, thereby reducing respondent burden.

A limitation of our study is the reliance on self-reported data and online data collection. Participants are not blinded to the study’s aims, which may influence their responses due to concerns about how their answers might be used. We recognize that the survey is lengthy, which may contribute to participant fatigue. To address this limitation, participants are provided with the option to save their responses and complete the study later. Another limitation includes factors outside the control of the research team that may affect timing for recruitment and data collection, such as the time taken to obtain ethics approval and data sharing agreements at collaborating sites. At the end of data collection, the sample size may be insufficient to include all subgroups in some psychometric tests (eg, DIF analyses by country or language). Finally, younger adolescents (aged 12‐14 y) and those assigned male at birth may be underrepresented in the sample, which may limit the generalizability of findings to these subgroups.

## Supplementary material

10.2196/100420 Multimedia Appendix 1 A priori construct validity hypotheses.

10.2196/100420 Peer Review Report 1 Granting agency peer review report.

## Abbreviations

2SLGBTQI+: Two-Spirit, lesbian, gay, bisexual, transgender, queer or questioning, and intersex
CIHR: Canadian Institutes of Health Research
COSMIN: Consensus-Based Standards for the Selection of Health Measurement Instruments
CTT: classical test theory
DIF: differential item functioning
GAC: gender-affirming care
ISPOR: The Professional Society for Health Economics and Outcomes Research
MID: minimally important difference
PROM: patient-reported outcome measure
RMT: Rasch measurement theory
SDC: smallest detectable change
STROBE: Strengthening the Reporting of Observational Studies in Epidemiology
TGD: transgender and gender diverse
TRT: test-retest
