# Supplementary material for: GENDER-Q Youth Patient-Reported Outcome Measure: Protocol for an International Cross-Sectional Field Test Study
Source: JMIR Res Protoc. 2026 Oct 5;15:e100420. doi: 10.2196/100420 (PMC13637991; doi:10.2196/100420)
Supplement: Supplementary file 1 [file resprot-v15-e100420-s002.pdf]

**Review Type/Type d'évaluation:** Committee Member 1/Membre de comité 1
**Name of Applicant/Nom du chercheur:** Klassen, Anne Frances
**Application No./Numéro de demande:** 427202
**Agency/Agence:** CIHR/IRSC
**Competition/Concours:** 2019-10-08 Chair: CIHR Sex and Gender Science Chair/Chaire: Chaire science des sexe et du genre des IRSC
**Committee/Comité:** Sex and Gender Science Chair/Chaire en science du sexe et du genre
**Title/Titre:** Developing a Patient-Reported Outcome Measure for Children and Youth Seeking Gender-Affirming Health Care (GENDER-Q Kids)

---

**Assessment/Évaluation:**

**Brief Summary of Proposal**

The specific aims of the chair are to develop and validate Patient Reported Outcome Measures or PROMs for trans and gender non-binary children and adolescents related to gender affirming health care; the research involves a qualitative conceptual development phase, which will include the development of candidate items for PROMs, which will then be followed by field testing the items and scales with a lrge international sample of children and youth seeking gender affirming care.

**Applicant Track Record**

The applicant has a strong track record of developing PROMs for a variety of health issues, and a highly productive career to date. The proposed chair builds on a recent Foundation grant to develop PROMs for adult transgender health care outcomes.

The researcher has only relatively recent experience working with trans populations, but does have some significant funding (Foundation grant) and is working with recognized international collaborations, with an important focus, and high likelihood of success.

**Research Program Proposal**

This is a strong well-written, clear proposal, with clear identification of the project aims and methods, as well as ways that it leverages important prior work. The proposed research has the potential to create important patient-focused clinical outcome measures for evaluating care,

The applicant has well-described international engagement and potential for wide-ranging international impact, solid cross-cultural approaches. Methodology is well-developed and clear, quite appropriate. The collaborating partners are an equally impressive international team.

The timeframe for the research is quite feasible, and aligns with the stated goals.

One area that is underdeveloped in the proposal is articulating how this work amplifies understanding of gender and/or sex—although the work is focused on transgender children and youth, there was minimal acknowledgment of the diversity of gender identities among trans and non-binary young people. It seems highly likely that one size will not fit all, and that specific PROMs will vary widely by gender identities, but there were no discussion of whether a PROM set might need to be tailored to specific gendered experiences, or gender groups. Indeed, there appeared to be an assumption that one could create a singular PROM that would be relevant for transfeminine and transmasculine and non-binary children and youth, without attending to the distinctly different gendered spaces they occupy. The gender and sex section of the application was perfunctory, and so this lack of discussion of gender within the proposal itself left me wondering to what extent this researcher had actually incorporated gender into prior PROM work, despite having some very specific primarily gendered health outcomes to develop PROMs around, such as the BREAST-Q. That reduces my enthusiasm somewhat for what is otherwise a very strong proposal.

| | |
|---|---|
| **Review Type/Type d'évaluation:** | Committee Member 1/Membre de comité 1 |
| **Name of Applicant/Nom du chercheur:** | Klassen, Anne Frances |
| **Application No./Numéro de demande:** | 427202 |
| **Agency/Agence:** | CIHR/IRSC |
| **Competition/Concours:** | 2019-10-08 Chair: CIHR Sex and Gender Science Chair/Chaire: Chaire science des sexe et du genre des IRSC |
| **Committee/Comité:** | Sex and Gender Science Chair/Chaire en science du sexe et du genre |
| **Title/Titre:** | Developing a Patient-Reported Outcome Measure for Children and Youth Seeking Gender-Affirming Health Care (GENDER-Q Kids) |

---

**Assessment/Évaluation:**

**Capacity Building Proposal**
The applicant has solid evidence of success in recruiting and mentoring a number of trainees from around the world, and helping launch their careers with effective co-authorship of a large number of papers. The current proposal builds on those successes by incorporating clinician scientists and graduate trainees, some with lived experience, in the research.

The applicant described a solid set of approaches to mentoring a large number of trainees in the highly effective methods, with extensive opportunities to develop important skills in conducting research, writing and presenting. There is high likelihood of success in building a further cadre of clinician scientists able to develop and evaluate outcome measures with pediatric patients for a variety of key health issues, although how the researcher would provide training to further their expertise in gender- and sex-based approaches to healthresearch, and thus advance the field, was not made explicit.

**Knowledge Translation Plan**
The KTE proposal is workmanlike, with little that is innovative, but there is a clear plan with a variety of strategies that are feasible and realistic approaches, and clearly the productivity is high.

**Environment and Support**
There is excellent evidence of support for this work, at a top tier research intensive university.

**Comments on the budget requested and a formal recommendation**.
No concerns about budget, well justified.

| | |
|---|---|
| **Review Type/Type d'évaluation:** | Committee Member 2/Membre de comité 2 |
| **Name of Applicant/Nom du chercheur:** | Klassen, Anne Frances |
| **Application No./Numéro de demande:** | 427202 |
| **Agency/Agence:** | CIHR/IRSC |
| **Competition/Concours:** | 2019-10-08 Chair: CIHR Sex and Gender Science Chair/Chaire: Chaire science des sexe et du genre des IRSC |
| **Committee/Comité:** | Sex and Gender Science Chair/Chaire en science du sexe et du genre |
| **Title/Titre:** | Developing a Patient-Reported Outcome Measure for Children and Youth Seeking Gender-Affirming Health Care (GENDER-Q Kids) |

**Assessment/Évaluation:**

**Applicant:**

The applicant is a highly productive, high-impact (h-index of 47-57), senior-career researcher in health outcomes (Professor of Pediatrics, Health Sciences at McMaster) with expertise in development of patient-reported outcome measures (PROMs). Applicant is CIHR Foundation Grant holder for a related research developing and refining the Gender-Q PROMs for transgender adults receiving gender affirming treatments, and also currently holds numerous project/operating grants as PI. Applicant has extensive experience with proposed methodology, and has clearly demonstrated success in the development, refinement, and dissemination of PROMs for various conditions where plastic surgeries are indicated, e.g., breast reconstruction, cleft palate. Only possible weakness is a relatively smaller involvement in student research supervision than is typical for a researcher at this career stage (graduate two PhDs, two MScs).

**Research Proposal:**

Proposed research to develop a PROMs for children and youth seeking gender affirming health care. The proposed methodology, qualitative item development followed by Rasch analysis, is well-established, empirically-supported, and highly rigorous and the applicant has extensive experience with this methodology. International field test of the measure will follow. Extensive and high-profile network of collaborators working on the adult Gender-Q. Chair award would capitalized on existing expertise, networks, infrastructure, and funding to develop patient outcome measure for a growing sector of health-care seeking children and youth.

**Capacity Building:**

Capacity building is focused on trainees, with funding to support two graduate students and two post-doctoral fellows. The training environment is excellent with significant opportunities to network with international leaders in patient-outcome research. Applicant has relatively limited student supervision experience.

**Knowledge Translation:**

The major highlight here is the free dissemination of the Gender-Kids PROMs to nonprofit users through the established website. Promotion of the measure is planned through academic channels.

**Environment and Support:**

The environment is exceptional and well-established with demonstrated success, supportive research infrastructure. Noted that the applicant is a member of CanChild centre of excellence with KT experts to assist.

| | |
|---|---|
| **Review Type/Type d'évaluation:** | Committee Member 2/Membre de comité 2 |
| **Name of Applicant/Nom du chercheur:** | Klassen, Anne Frances |
| **Application No./Numéro de demande:** | 427202 |
| **Agency/Agence:** | CIHR/IRSC |
| **Competition/Concours:** | 2019-10-08 Chair: CIHR Sex and Gender Science Chair/Chaire: Chaire science des sexe et du genre des IRSC |
| **Committee/Comité:** | Sex and Gender Science Chair/Chaire en science du sexe et du genre |
| **Title/Titre:** | Developing a Patient-Reported Outcome Measure for Children and Youth Seeking Gender-Affirming Health Care (GENDER-Q Kids) |

**Assessment/Évaluation:**

**Budget:**

Participant stipends for qualitative interviews is entered twice, so -$4K. Otherwise, no concerns.

**Review Type/Type d'évaluation:** Committee Member 3/Membre de comité 3
**Name of Applicant/Nom du chercheur:** Klassen, Anne Frances
**Application No./Numéro de demande:** 427202
**Agency/Agence:** CIHR/IRSC
**Competition/Concours:** 2019-10-08 Chair: CIHR Sex and Gender Science Chair/Chaire: Chaire science des sexe et du genre des IRSC
**Committee/Comité:** Sex and Gender Science Chair/Chaire en science du sexe et du genre
**Title/Titre:** Developing a Patient-Reported Outcome Measure for Children and Youth Seeking Gender-Affirming Health Care (GENDER-Q Kids)

---

**Assessment/Évaluation:**

The applicant has the required expertise and achievements record.
The applicant proposes developing a GENDER-Q for pediatric patients. The proposal includes more data on previous PROMs than the methods used to develop the new one.
Timeline is missing.
KT strategy is a strength.
A disclosure on budgetary overlaps is required. Also the PI’s total time allocated for this chair to finalize the objectives in 4 years.
Budget can be improved (The amount allocated to personnel vs trainees/capacity building)

**Review Type/Type d'évaluation:** SO Notes /Notes de l'agent scientifique
**Name of Applicant/Nom du chercheur:** Klassen, Anne Frances
**Application No./Numéro de demande:** 427202
**Agency/Agence:** CIHR/IRSC
**Competition/Concours:** 2019-10-08 Chair: CIHR Sex and Gender Science Chair/Chaire: Chaire science des sexe et du genre des IRSC
**Committee/Comité:** Sex and Gender Science Chair/Chaire en science du sexe et du genre
**Title/Titre:** Developing a Patient-Reported Outcome Measure for Children and Youth Seeking Gender-Affirming Health Care (GENDER-Q Kids)

---

**Assessment/Évaluation:**

## Strengths:

- The objective of this program is to develop and validate gender affirming Patient Reported Outcome Measures or PROMs for pediatric patients. The applicant has significant experience in developing PROMs for a variety of health issues and the proposed methodology.
- The applicant has had a productive career to date. The researcher has significant funding and recent experience with transgender individuals
- This was a well written and clear proposal that will produce an important clinically focused measure. They provide solid cross-cultural approaches and have assembled strong collaborations.
- The applicant has a strong history of trainee mentorship and promotion. This is an excellent environment for the development of trainees.
- excellent environmental support.

## Weaknesses:

- There was minimal acknowledgement of the diversity of gender minorities.
- They treat the PROMS measure with assumptions of the homogeneity of outcomes and this was viewed a shortcoming of the grant. The applicants could proactively deal with this diversity in the proposal and could address how these PROMS could be tailored to specific trans and non-binary groups.
- the KT proposal was clearly defined but was not necessarily innovative.
- It was acknowledged that there was youth engagement in the development of the PROMS. However, there was not youth engagement in conduct and direction of the research and this was perceived as a weakness.

## Budget:

No issues discussed – one noted in a reviewer comment.
