# Supplementary material for: GENDER-Q Youth Patient-Reported Outcome Measure: Protocol for an International Cross-Sectional Field Test Study
Source: JMIR Res Protoc. 2026 Oct 5;15:e100420. doi: 10.2196/100420 (PMC13637991; doi:10.2196/100420)
Supplement: Supplementary file 2 [file resprot-v15-e100420-s001.docx]

**Multimedia Appendix 1: A priori construct validity hypotheses**

|  | **Body Image** | **Psych Distress** | **Psych WB** | **Life Impact** | **Family** | **School** | **Social** | **Teacher** | **Voice Sound** | **Voice Distress** | **Binding WB** | **Binding AE** | **Tucking AE** | **Health Care Team** | **Info Hormones** | **Treatment Outcome** |
| --- | --- | --- | --- | --- | --- | --- | --- | --- | --- | --- | --- | --- | --- | --- | --- | --- |
| **GROUP HYPOTHESES: SCORES HIGHER FOR THE FOLLOWING:** | | | | | | | | | | | | | | | | |
| 1. Those who live (vs. do not) in their gender identity | ✓ | ✓ | ✓ | ✓ | ✓ |  |  |  |  |  |  |  |  |  |  |  |
| 2. Those who like (vs. do not) how their face looks overall | ✓ |  |  |  |  |  |  |  |  |  |  |  |  |  |  |  |
| 3. Those not teased or bullied (vs. are) in past month |  | ✓ | ✓ |  |  | ✓ | ✓ |  |  |  |  |  |  |  |  |  |
| 4. Those in a school that support (vs. do not) TGD students |  |  |  |  |  | ✓ |  | ✓ |  |  |  |  |  |  |  |  |
| 5. Those happy (vs. not happy) with their teachers at school |  |  |  |  |  | ✓ |  | ✓ |  |  |  |  |  |  |  |  |
| 6. Those who rate their mental health higher (poor to excellent) |  | ✓ | ✓ | ✓ |  |  |  |  |  |  |  |  |  |  |  |  |
| 7. Those who feel happy (vs. sad) |  | ✓ | ✓ | ✓ |  |  |  |  |  |  |  |  |  |  |  |  |
| 8. Those happy (vs. not happy) with their life overall |  | ✓ | ✓ | ✓ |  |  | ✓ |  |  |  |  |  |  |  |  |  |
| 9. Those with ≥1 close friend (vs. none) |  |  |  |  |  |  | ✓ |  |  |  |  |  |  |  |  |  |
| 10. Those with ≥1 close friend (vs. none) who knows they are TGD |  |  |  |  |  |  | ✓ |  |  |  |  |  |  |  |  |  |
| 11. Those who spend time (vs. do not) with friends |  |  |  |  |  |  | ✓ |  |  |  |  |  |  |  |  |  |
| 12. Those happy (vs. not) with their social life |  |  |  | ✓ |  |  | ✓ |  |  |  |  |  |  |  |  |  |
| 13. Those who rate their social life higher (poor to excellent) |  |  |  | ✓ |  |  | ✓ |  |  |  |  |  |  |  |  |  |
| 14. Those happy (vs. not happy) with their family life |  |  |  |  | ✓ |  |  |  |  |  |  |  |  |  |  |  |
| 15. Those living with ≥1 person (vs. none) who supports their TGD identity |  |  |  |  | ✓ |  |  |  |  |  |  |  |  |  |  |  |
| 16. Those living with supportive family (vs. not) of their TGD identity |  |  |  |  | ✓ |  |  |  |  |  |  |  |  |  |  |  |
| 17. Those happy (vs. not happy) with how they look overall | ✓ | ✓ | ✓ | ✓ |  |  |  |  |  |  | ✓ |  |  |  |  |  |
| 18. Those who voice sounds right (vs. too high or too low) |  |  |  |  |  |  |  |  | ✓ | ✓ |  |  |  |  |  |  |
| 19. Those happy (vs. not happy) with how their voice sounds |  |  |  |  |  |  |  |  | ✓ | ✓ |  |  |  |  |  |  |
| 20. Those who like (vs. dislike) how their voice sounds overall |  |  |  |  |  |  |  |  | ✓ | ✓ |  |  |  |  |  |  |
| 21. Those happy (vs. not happy) with how they look when they bind | ✓ |  |  |  |  |  |  |  |  |  | ✓ |  |  |  |  |  |
| 22. Those whose chest looks completely flat (vs. not) when they bind | ✓ |  |  |  |  |  |  |  |  |  | ✓ |  |  |  |  |  |
| 23. Those who bind fewer days/hours in a week |  |  |  |  |  |  |  |  |  |  |  | ✓ |  |  |  |  |
| 24. Those with did not see (vs. did see) HCT for binding problems |  |  |  |  |  |  |  |  |  |  |  | ✓ |  |  |  |  |
| 25. Those without (vs. with) a binding problem |  |  |  |  |  |  |  |  |  |  |  | ✓ |  |  |  |  |
| 26. Those who tuck fewer days/hours in a week |  |  |  |  |  |  |  |  |  |  |  |  | ✓ |  |  |  |
| 27. Those without (vs. with) a tucking problem |  |  |  |  |  |  |  |  |  |  |  |  | ✓ |  |  |  |
| 28. Those who did not see (vs. did see) HCT for tucking problems |  |  |  |  |  |  |  |  |  |  |  |  | ✓ |  |  |  |
| 29. Those on longer duration of hormone treatment | ✓ |  |  |  |  |  |  |  |  |  |  |  |  | ✓ | ✓ | ✓ |
| 30. Those happy (vs. not happy) with treatment in past month |  |  |  |  |  |  |  |  |  |  |  |  |  | ✓ |  | ✓ |
| 31. Those who do not want (vs. want) top surgery | ✓ |  |  |  |  |  |  |  |  |  |  |  |  |  |  |  |
| 32. Those who do not want (vs. want) voice therapy |  |  |  |  |  |  |  |  | ✓ | ✓ |  |  |  |  |  |  |
| 33. Those happy (vs. not happy) with the staff at gender clinic |  |  |  |  |  |  |  |  |  |  |  |  |  | ✓ |  | ✓ |
| 34. Those with more visits (vs. less) to the gender clinic |  |  |  |  |  |  |  |  |  |  |  |  |  | ✓ |  |  |
| 35. Those happy (vs. not happy) with knowledge of hormones |  |  |  |  |  |  |  |  |  |  |  |  |  |  | ✓ |  |
| 36. Those who know more (vs. less) about hormones |  |  |  |  |  |  |  |  |  |  |  |  |  |  | ✓ |  |
| **GROUP HYPOTHESES TOTAL** | **7** | **6** | **6** | **7** | **4** | **3** | **7** | **2** | **4** | **4** | **3** | **3** | **3** | **4** | **3** | **3** |
| **CORRELATIONAL HYPOTHESES: see footnote at the bottom of the table to interpret magnitude of association** | | | | | | | | | | | | | | | | |
| 37. Positive correlation Body Image |  | 3 | 3 | 3 |  |  | 2 |  |  |  | 1 |  |  |  |  | 2 |
| 38. Positive correlation Psych Distress | 3 |  | 3 | 3 | 1 | 1 | 3 | 1 | 2 | 2 |  |  |  |  |  | 2 |
| 39. Positive correlation Psych Well-being | 3 | 3 |  | 3 | 1 | 1 | 3 | 1 | 2 | 2 |  |  |  |  |  | 2 |
| 40. Positive correlation Life Impact | 3 | 3 | 3 |  | 1 | 1 | 3 | 1 | 2 | 2 |  |  |  |  |  | 2 |
| 41. Positive correlation Family |  | 1 | 1 | 1 |  |  |  |  |  |  |  |  |  |  |  |  |
| 42. Positive correlation School |  | 1 | 1 | 1 |  |  |  | 1 |  |  |  |  |  |  |  |  |
| 43. Positive correlation Social | 2 | 3 | 3 | 3 |  |  |  |  | 2 | 2 |  |  |  |  |  |  |
| 44. Positive correlation Teacher |  | 1 | 1 | 1 |  | 1 |  |  |  |  |  |  |  |  |  |  |
| 45. Positive correlation Voice Sound |  | 2 | 2 | 2 |  |  | 2 |  |  | 3 |  |  |  |  |  | 1 |
| 46. Positive correlation Voice Distress |  | 2 | 2 | 2 |  |  | 2 |  | 3 |  |  |  |  |  |  | 1 |
| 47. Positive correlation Binding Well-being | 1 |  |  |  |  |  |  |  |  |  |  |  |  |  |  |  |
| 48. Positive correlation Binding Adverse Effects |  |  |  |  |  |  |  |  |  |  |  |  |  |  |  |  |
| 49. Positive correlation Tucking Adverse Effects |  |  |  |  |  |  |  |  |  |  |  |  |  |  |  |  |
| 50. Positive correlation Health Care Team |  |  |  |  |  |  |  |  |  |  |  |  |  |  | 1 | 1 |
| 51. Positive correlation Information: Hormones |  |  |  |  |  |  |  |  |  |  |  |  |  | 1 |  | 1 |
| 52. Positive correlation Treatment Outcome | 2 | 2 | 2 | 2 |  |  |  |  | 1 | 1 |  |  |  | 1 | 1 |  |
| **CORRELATIONAL HYPOTHESES TOTAL** | **6** | **10** | **10** | **10** | **3** | **4** | **6** | **4** | **6** | **6** | **1** | **0** | **0** | **2** | **2** | **8** |
| **TOTAL (GROUP + CORRELATIONAL)** | **14** | **16** | **16** | **17** | **7** | **7** | **14** | **6** | **10** | **10** | **4** | **3** | **3** | **6** | **5** | **11** |

AE = Adverse Effects; WB = Well-being; HCP = Health Care Team; 1=>0.3; 2=0.3-0.5; 3=>0.5. Note: 2 and 3 are based on correlations between scales from the GENDER-Q adult module analysis
